# A modified decision tree approach to improve the prediction and mutation discovery for drug resistance in *Mycobacterium tuberculosis*

**DOI:** 10.1186/s12864-022-08291-4

**Published:** 2022-01-11

**Authors:** Wouter Deelder, Gary Napier, Susana Campino, Luigi Palla, Jody Phelan, Taane G. Clark

**Affiliations:** 1 London School of Hygiene & Tropical Medicine, Keppel Street, London, WC1E 7HT UK; 2 Dalberg Advisors, 7 Rue de Chantepoulet, CH-1201 Geneva, Switzerland; 3 Department of Public Health and Infectious Diseases, University of Rome La Sapienza, Rome, Italy; 4 Department of Infection Biology, Faculty of Infectious and Tropical Diseases, London School of Hygiene and Tropical Medicine, London, UK

**Keywords:** *Mycobacterium tuberculosis*, Ethionamide, Cycloserine, PAS, Drug resistance, Machine learning

## Abstract

**Background:**

Drug resistant *Mycobacterium tuberculosis* is complicating the effective treatment and control of tuberculosis disease (TB). With the adoption of whole genome sequencing as a diagnostic tool, machine learning approaches are being employed to predict *M. tuberculosis* resistance and identify underlying genetic mutations. However, machine learning approaches can overfit and fail to identify causal mutations if they are applied out of the box and not adapted to the disease-specific context. We introduce a machine learning approach that is customized to the TB setting, which extracts a library of genomic variants re-occurring across individual studies to improve genotypic profiling.

**Results:**

We developed a customized decision tree approach, called Treesist-TB, that performs TB drug resistance prediction by extracting and evaluating genomic variants across multiple studies. The application of Treesist-TB to rifampicin (RIF), isoniazid (INH) and ethambutol (EMB) drugs, for which resistance mutations are known, demonstrated a level of predictive accuracy similar to the widely used TB-Profiler tool (Treesist-TB vs. TB-Profiler tool: RIF 97.5% vs. 97.6%; INH 96.8% vs. 96.5%; EMB 96.8% vs. 95.8%). Application of Treesist-TB to less understood second-line drugs of interest, ethionamide (ETH), cycloserine (CYS) and para-aminosalisylic acid (PAS), led to the identification of new variants (52, 6 and 11, respectively), with a high number absent from the TB-Profiler library (45, 4, and 6, respectively). Thereby, Treesist-TB had improved predictive sensitivity (Treesist-TB vs. TB-Profiler tool: PAS 64.3% vs. 38.8%; CYS 45.3% vs. 30.7%; ETH 72.1% vs. 71.1%).

**Conclusion:**

Our work reinforces the utility of machine learning for drug resistance prediction, while highlighting the need to customize approaches to the disease-specific context. Through applying a modified decision learning approach (Treesist-TB) across a range of anti-TB drugs, we identified plausible resistance-encoding genomic variants with high predictive ability, whilst potentially overcoming the overfitting challenges that can affect standard machine learning applications.

**Supplementary Information:**

The online version contains supplementary material available at 10.1186/s12864-022-08291-4.

## Introduction

Tuberculosis (TB), caused by *Mycobacterium tuberculosis*, is a pressing global health problem, with > 10 million cases and 1.4 million associated deaths in 2019 [1]. First-line TB treatment uses combinations of the drugs rifampicin (RIF), isoniazid (INH), ethambutol (EMB) and pyrazinamide (PZA) [2]. Drug-resistance requires switching to second-line therapies combined in customized treatment protocols, which might include fluoroquinolones and injectable drugs, as well as ethionamide (ETH), cycloserine (CYS) and para-aminosalisylic acid (PAS), among others. Historically, a cascade of resistance has been defined, from resistance to RIF (RR-TB), to additional resistance to INH leading to multidrug resistance (MDR-TB), further leading to an extensively drug resistant (XDR-TB) class that is MDR-TB with additional resistance to fluoroquinolones and second-line injectables. Recently, there was a new definition of pre-XDR (MDR-TB and resistance to any fluoroquinolone) and an updated definition of XDR-TB (pre-XDR and resistance to least one additional Group A drug, including levofloxacin or moxifloxacin, bedaquiline and linezolid) [3]. These updates provide a framework for increasing progression of the severity of disease linked to resistance to additional anti-TB drugs [3].

The mechanisms that cause *M. tuberculosis* drug resistance are linked to genomic variants in drug targets or pro-drug activators, including single nucleotide polymorphisms (SNPs) and small insertions and deletions (indels), some occurring in gene-gene interactions. Pro-drug activators convert mycobacterial enzymes that convert pro-drugs, such as INH and ETH, into their active form. If these enzymes (e.g., catalase peroxidase (KatG) for INH) are not essential, their coding genes can acquire mutations such as frameshifts which lead to loss of function, and consequently, the respective drug is not converted and resistance is caused. However, not all resistance mechanisms are well understood [4–6], especially for second-line drugs (e.g. PAS). Drug-resistance has been traditionally assessed through bacterial culture-based phenotypic drug susceptibility testing (DST), which can be time-consuming and resource intensive, with reproducibility and inhibitory concentration cut-off challenges for particular drugs [7]. Whole-genome sequencing (WGS) offers an alternative approach to infer resistance through the identification of associated genomic mutations [8], called “genotypic resistance” profiling. TB-Profiler software [9, 10] uses a curated library of > 1000 mutations to predict genotypic resistance across 14 anti-TB drugs. The use of WGS can reaffirm known resistance mutations and uncover new candidates through genome-wide association studies (GWAS) and convergent evolution analysis [11]. However, GWAS approaches typically focus on single variants at a time in regression models, whereas resistance phenotype prediction from WGS is a classification problem with high-dimensional input and potential complex interactions, a standard task in machine learning [12]. Therefore, the ongoing generation of large datasets using WGS is highly suited to the application of machine learning methods to improve “genotypic resistance” profiling [12].

The application of machine learning methods to *M. tuberculosis* has shown some impressive performances in genotypic profiling [13–17]. However, these models have several drawbacks that could affect their application in clinical settings, including their interpretability and an optimism bias related to the inclusion of non-associated cross-resistance and bacterial lineage markers; both leading to reduced predictive performance in hospital and other clinical settings [15]. The performance of machine learning models has also been relatively poor for a subset of second-line drugs (CYS, PAS, ETH), which in general are less often studied and analysed [11, 15]. The generally lower performance for CYS, PAS and ETH suggests that mechanisms of resistance are less well understood, and that potentially rare alleles are being missed and excluded from models [15]. Our study aims to attempt to detect new genomic variants that might cause resistance for CYS, PAS, and ETH. The approach involves a customized (decision tree) machine learning algorithm, called Treesist-TB*,* which detects genomic variants in individual studies within the aggregated datasets, and can model variant interactions. It attempts to be robust to the presence of DST errors in some of the individual studies, which can lead to genomic variants being undetected in the analysis of the aggregate dataset.

## Results

### Genomic and phenotypic data

WGS data was available for 32,689 (32 k) *M. tuberculosis* samples, which covered the main lineages 1 (9.6%), 2 (25.2%), 3 (11.4%) and 4 (51.0%) (S1 Table). Most samples were pan-susceptible (77.9%), but RR-TB (1.3%), MDR-TB (13.0%) and XDR-TB (2.3%) phenotypes were also represented. Phenotypic DST data was not available for all isolates, with limited data generation for PAS (*n* = 1114, 8.8% resistant), CYS (*n* = 833, 18.0% resistant), and ETH (*n* = 2138, 32.2% resistant) (S2 Table; Table 1), as these drugs are mostly prescribed to and assessed in patients with RR-TB and MDR-TB.

**Table 1:** Predictive performance across algorithms

Drug	Total tests	% resistance	TB-Profiler				Treesist-TB^**a**^			
			Sens	Spec	Acc	AUC	Sens	Spec	Acc	AUC
INH	1835	16.2	86.2	98.4	96.5	92.3	84.2	99.2	96.8	91.7
RIF	2045	8.1	90.3	98.2	97.6	94.2	86.1	98.5	97.5	92.3
EMB	1999	3.5	71.4	96.7	95.8	84.1	57.1	98.2	96.8	77.7
PAS	1114	8.8	38.8	95.7	90.7	67.2	64.3	90.6	88.2	77.4
CYS	833	18.0	30.7	95.2	83.6	62.9	45.3	93.7	85.0	69.5
ETH	2118	32.2	71.1	78.6	76.2	74.8	72.1	75.8	74.6	73.9
			***Regular Decision Tree***				**Single optimized Tree**^**b**^			
			**Sens**	**Spec**	**Acc**	**AUC**	**Sens**	**Spec**	**Acc**	**AUC**
INH	1835	16.2	85.6	100	97.7	92.9	80.2	99.2	96.1	89.8
RIF	2045	8.1	81.2	100	98.5	91.5	87.3	99.8	98.8	93.6
EMB	1999	3.5	32.9	99.7	97.3	82.9	34.3	99.5	97.2	83
PAS	1114	8.8	64.3	100	96.9	85.5	50	97.8	93.6	74.1
CYS	833	18.0	33.3	99.4	87.5	67.3	35.3	98	86.7	66.7
ETH	2118	32.2	48.8	94.3	79.7	77.5	49.6	92.5	78.7	76.2

*INH* Isoniazid, *RIF* Rifampicin, *PAS* para-aminosalisylic acid, *CYS* cycloserine, *ETH* ethionamide, *EMB* Ethambutol, *Sens* Sensitivity, *Spec* Specificity, *Acc* Accuracy, *AUC* Area under the ROC Curve

^a^default application of Treesist-TB

^b^application of Treesist-TB with a single combined study dataset

### Application of Treesist-TB to first-line drugs

Treesist-TB is a python-based machine learning algorithm that fits customized decision trees across individual studies and combines the extracted features to make final resistance predictions. It can also, if desired, be run assuming all data is from a single study (referred to as a “single optimised tree”). The algorithm was first applied to well-understood first-line drugs, using a subset of isolates that had complete DSTs (RIF: *n* = 2045, 8.1% resistant, 7 studies; INH *n* = 1835, 16.2% resistant; 6 studies; EMB: *n* = 1999, 3.5% resistant, 5 studies; S2 Table) across second-line drugs.

We fitted a default Treesist-TB tree assuming individual studies, as well as, for comparison purposes, single optimised and regular decision trees. The single optimized trees were simpler and contained fewer implausible sub-structures than regular decision trees (S2 Figure) while maintaining relevant structures such as double mutations and gene-gene interactions. In particular, the optimized trees contain fewer genes (INH: 27 vs. 5; RIF: 6 vs. 4; EMB: 5 vs. 4) but generally more individual variants (INH: 29 vs. 6; RIF: 15 vs. 20; EMB: 6 vs. 5) than regular decision trees. However, single optimized trees do include some unlikely features that might arise from overfitting on DST errors or other artefacts in the aggregated dataset (S2 Figure), so we applied the default Treesist-TB algorithm, which incorporates information from individual sub-studies.

The Treesist-TB algorithm identified several predictive genomic variants for resistance of RIF (*n* = 20; 7 unreported in the TB-Profiler library), INH (*n* = 20, 13 unreported) and EMB (*n* = 10, 2 unreported) (S1 Figure, S2 Figure, Table 2; S4 Table). These included mutations in established loci such as *rpoB* (*n* = 18, RIF), *katG* (*n* = 17, INH), and *embB* (*n* = 7, EMB). A confirmation analysis of the Treesist-TB mutations in the set of validation isolates (*n* = ~ 30 k of 32 k, not analysed by Treesist-TB)*,* revealed that none were present in susceptible strains, but they were frequent in both MDR-TB (median (maximum): RIF 1.6% (65.3%); INH < 0.1% (79.2%); EMB 2.1 (23.8)) and XDR-TB (RIF 0.8% (70.7%); INH < 0.1% (78.6%); EMB 3.1% (35.3%)) isolates. The predictive accuracy of resistance from Treesist-TB was similar to the TB-Profiler tool (Treesist-TB vs. TB-Profiler: RIF 97.5% vs. 97.6%; INH 96.8% vs. 96.5%; EMB 96.8% vs. 95.8%), and like those from the single optimized and regular decision trees (Table 1), whose models include mutations not associated with resistance.

**Table 2:** The Treesist-TB inferred variants

Drug	Gene	# variants in the 32 k dataset*	Treesist-TB Mutations**
RIF	*rpoB*	757	**N163K**, V170F, L430P, Q432K, Q432L, D435Y, D435V, S441L, **H445D, H445D, H445N**, H445Y, **H445R,** H445L, S450L, L452P, I491F
RIF	*rpoC*	700	**N1239D, Q1289A**
INH	*ahpC*	31	**-57C > T, −48G > A**
INH	*fabG1*	26	**-126G > A**
INH	*katG*	648	**Y597D, T568P, A476V,** S315T, S315N, S302R, **W300C**, G297V, **P193fs, L159F**, **G156D, A144V, D142G,** L141F, N138D, A109V, **Y98C**
EMB	*embA*	743	**-31delC,** −16C > T, **−16C > A**
EMB	*embB*	762	M306V, M306L, M306I, G406A, Q497K, Q497R, D1024N
PAS	*Rv2670c*	191	**A5V**
PAS	*folC*	262	Q153G, Q153A, S150G, **S98G, R49Q,** I43T
PAS	*thyX*	148	**-4C > T, −9G > A**, −16C > T, **−18G > T**
CYS	*alr*	239	**Y388D, L283P,** L113R, **T20M**
CYS	*rpoC*	700	**D485Y**, I491T
ETN	*ethA*	494	W455*, K448fs, P436fs, A352fs, P334A, F320S, L295fs, C294*, R279*, Q269*, M260I, W256*, C253F, T236fs, Y235fs, W228*, N226fs, K224*, A222V, S208L, R207G, V202F, L194P, T186P, P164R, P160fs, C137R, C137R, W116*, K103fs, W45*, K37fs, L35R, Q24*, D6fs
ETN	*fabG1*	26	**-118C > G, −34C > T,** **−15C > T, −8 T > C,** **−8 T > A**
ETN	*gyrA*	764	A90V, S91P, D94A, D94G
ETN	*inhA*	108	I21T, **R27W,** I194T, **P251R**
ETN	*mshA*	250	**A133fs, H175fs, V237L, A422V**

* 32 k *M. tuberculosis* isolates [18]

** **Bolded** if not in TB-Profiler in https://github.com/jodyphelan/tbdb/blob/master/tbdb.csv; * stop codon

*INH* Isoniazid, *RIF* Rifampicin, *PAS* para-aminosalisylic acid, *CYS* cycloserine, *ETH* ethionamide, *EMB* Ethambutol

** Mutations underlined if they are in > 5% of MDR-TB or XDR-TB strains in the 32 k *M. tuberculosis* isolates

### Application to selected second-line drugs

Given the strong performance for first-line drugs, Treesist-TB was then implemented for PAS, CYS and ETH, for which predictive accuracy has historically been poor and resistance mutations are only partially known [10]. Again, for comparison purposes, we fitted a single optimized tree for each drug and contrasted the performance and structure with regular classification trees (S3 Figure; Table 2). The results revealed that the optimized trees contain both fewer genes (PAS: 33 vs. 4; CYS: 7 vs. 3; ETH: 11 vs. 3) and variants (PAS: 37 vs. 7; CYS: 7 vs. 3; ETH: 13 vs. 5) than regular decision trees. The single optimized trees were simpler and contained fewer implausible sub-structures than regular decision trees, which appeared to be over-fitted (S2 Figure).

For PAS, the default application of Treesist-TB detected 11 genomic variants across three genes (*folC* 6*, Rv2670c* 1, *thyX 4*) (Table 2). Six of the variants are unreported in TB-Profiler, occurring in *folC* (R49Q, Ser98G), *Rv2670c* (A5V), and *thyX* (three indels: -4C > T, −9G > A, −18G > T) (S5 Table). These PAS mutations were present in XDR-TB samples in the validation set (frequency: median 0.2%, max. 6.1%) (S5 Table). For PAS, compared to TB-Profiler, the Treesist-TB mutation set leads to a higher sensitivity (64.3% vs. 38.8%), lower specificity (90.6% vs. 95.7%) and similar overall accuracy (88.2% vs. 90.7%) for drug resistance prediction (Table 1). For CYS, Treesist-TB identified six variants across two genes (*rpoC 2,* 1 unreported; *alr* 4, 3 unreported). *RpoC* is a locus linked to compensatory effects in RIF resistance. The CYS mutations were present in XDR-TB samples in the validation set (frequency: median < 0.1, max. 8.5%) (S5 Table). Compared to TB-Profiler, the set of Treesist-TB mutations had a higher sensitivity (45.3% vs. 30.7%), and similar specificity (93.7% vs. 95.2%) and overall accuracy (85.0% vs. 83.6%) for resistance prediction. For ETH, Treesist-TB identified 52 genomic variants, more than half in *ethA* (35; 67.3%), with others found across four genes (*inhA* 4, *gyrA* 4*, mshA* 4, *fabG1 promoter* 5). Most variants are not present in the TB-Profiler library (*ethA* 34, *inhA* 2, *mshA* 4, *fabG1 promoter* 5). *EthA, fabG1 promoter* and *inhA* are established ETH related loci, but *gyrA* is linked to fluoroquinolone resistance, and *mshA* is known to encode a glycosyl-transferase enzyme involved in mycothiol biosynthesis that can affect ETH activation. These mutations for ETH were present in XDR-TB samples in the validation set (frequency: median < 0.1%, max. 36.5%) (S5 Table). For ETH, compared to TB-Profiler, Treesist-TB has a marginally higher sensitivity (72.1% vs. 71.1%), lower specificity (75.8% vs. 78.6%) and a similar overall accuracy (74.6% vs. 76.2%) for drug resistance prediction.

## Discussion

The relatively poor knowledge of underlying mutations for second-line anti-TB drug resistance will make prospects for WGS-informed clinical and infection control more difficult. Whilst machine learning has the promise to fill any gaps in “genetic” knowledge, some implementations for *M. tuberculosis* “genotypic profiling” have led to over-optimistic predictive abilities and models with mutations that are not biologically plausible or unrelated to the resistance of interest. Our work describes a decision tree machine learning approach, called Treesist-TB, which attempts to account for inter-study differences and constrains the size of models, thereby minimising the risk of over-fitting due to phylogenetic or false resistance-associated mutations. Its application to RIF, INH and EMB drugs, with known resistance mechanisms, detected both established and unreported mutations in functional pathways, and had predictive abilities similar to other machine learning implementations and the TB-Profiler tool. Application of Treesist-TB to CYS, PAS and ETH drugs, whose underlying resistance variants are less established and are less often studied, detected putative non-synonymous SNPs and frameshift mutations in activation pathways. For the PAS drug, genomic variants were found in the *folC* gene, which interrupts bioactivation within the folate biosynthetic pathway [19]. Similarly, mutations were found in the *alr* gene encoding alanine racemase that compensates for the inhibitory effect of CYS [20]. Finally, for ETH, the majority of mutations were detected in the *ethA* gene that activates ETH by the NADPH-specific flavin adenine dinucleotide-containing monooxygenase EthA [21]. Importantly, integrated WGS and DST studies for relatively new anti-TB drugs (e.g., bedaquiline, clofazimine and delamanid) are much-needed, as current low sample sizes make the determination of mutations underlying their resistance difficult [22].

Treesist-TB detects SNPs by working with the largest datasets possible, where some of the reported performance problems for second-line drugs are due to the exclusion of rare alleles. More importantly, Treesist-TB considers individual sub-studies that make up the large dataset, implicitly adjusting for potential DST or mislabelling errors in individual studies, which are potentially more common in some laboratories or drug assays. Treesist-TB also incorporates existing knowledge on which sub-structures in the decision trees are biologically less plausible, such as reversion mutations, and can prune these structures. If required, the approach can give preference to known resistance genomic variants in tree model building and control its complexity by placing a ceiling on the number of previously unknown resistance mutations. In this sense, Treesist-TB can take advantage of prior knowledge and insights specific to TB drug resistance, thereby providing a counterweight against the increasing usage of machine learning “out of the box”, which can lead to models that do not generalize well in clinical practice.

Our analysis revealed that standard machine learning approaches could, even after regular cross-validation, overfit in subtle manners that lead to an upward bias in performance and not translate into a high out-of-training-set performance. Although, a robust simulation study that considers a number of machine learning approaches is beyond the scope of our work, previous studies have shown that some implementations have boosted performance through the selection of cross-resistance markers that are unlikely to be causally related to resistance to the drug under investigation [15]. These unrelated markers might get selected as features by machine learning models due to the unique structure of TB datasets, including arising from *M. tuberculosis* phylogenetic structures and sequential drug testing practices. Similarly, fitted tree structures with features that are biologically unrelated to resistance might lead to impressive performance within the training set, but may be inappropriate for predictions in clinical practice. These problems will be exacerbated for more complex models that have a greater number of parameters, such as convolutional neural nets [23].

## Conclusions

In general, with the increasing application of WGS data in a clinical or research setting, there is a need for robust and interpretable machine learning models that take advantage of the resulting large and growing datasets, whilst being robust to data errors. One important application is in antimicrobial resistance (AMR) genotypic profiling, which could ultimately replace phenotypic DST approaches. However, any AMR models derived must be reliable in terms of prediction, generalize across clinical settings, and adapt to increasing data and knowledge. In addition, such models need to account for the idiosyncrasies of pathogens and infections, where *M. tuberculosis* is highly clonal and has no horizontal gene transfer, but for other pathogens there may be plasmid derived AMR. In conclusion, we have developed Treesist-TB*,* which can assist with identifying mutations and prediction drug resistance in a TB context. Through providing software for its implementation, the utility beyond TB can be evaluated, and the approach potentially refined for other AMR settings.

## Materials and methods

### Phenotypic and sequencing data

The main dataset consists of 32,689 (32 k) isolates with whole genome sequencing (WGS) and phenotypic drug susceptibility test (DST) data (see S1 Table [18];). The laboratory DST followed WHO recommended protocols and practice (see [11]). XDR-TB was defined using the recently replaced definition, that is, being MDR-TB with additional resistance to fluoroquinolones and second-line injectables. This is because the isolates were collected, processed, and resistance patterns interpreted for treatment options before the new definitions were introduced [3]. DST data was not available for every isolate across all drugs, as only those individuals resistant to first-line treatments are typically tested for second-line resistance. All isolates with PAS, CYS and ETH DST were included in the analysis (see S2 Table for sample sizes). A subset with complete INH, RIF and EMB DST data and with similar characteristics in terms of sample size and number of individual studies were chosen for Treesist-TB benchmark analysis (S2 Table). The residual 31 k isolates were used for validation through the analysis of mutation frequencies across susceptible and resistance groups. The raw sequence data were mapped to the H3Rv reference genome using *bwa-mem* software, and genomic variants (SNPs, indels) were called from the consensus of GATK and *samtools* software. Most genomic variants (98.9%) have low minor allele frequencies (< 1%), and we excluded SNPs in hypervariable PE/PPE gene families and with synonymous mutations (see [18]).

### Treesist-TB model

The Treesist-TB model is a major extension of a simple decision tree approach (sklearn implementation, v0.23.1) with the following modifications: (1) incorporation of prior parameters on which features to prioritize in the tree building in case of ties; 2) incorporation of tree pruning to limit interactions in the tree that are *a priori* determined to be unlikely (e.g. double mutations that compensate resistant mutations and restore drug sensitivity); (3) incorporation of prior parameters for the maximum number of genes (not genomic variants) in a tree. Although Treesist-TB is compatible with regular cross-validation methods (e.g., leave k-fold out), these approaches may lead to unstable results for trees in general. To prevent trees from having excessive depth, the setting of priors for the maximum number of new genes outside known resistance genes (not variants) has been implemented. We extracted a set of genomic variants using a consensus rule that variants were only included when in genes that were more than once detected across sub-datasets (S1 Figure).

### Model fitting

The predictive performance of the final models fitted to the entire dataset was measured using sensitivity, specificity, accuracy, and area under the ROC curve (AUC) metrics, assuming DST results as the gold standard. We compared the performance of the (default) Treesist-TB model primarily with the TB-Profiler software and mutation library (> 1000 SNPs, indels or large deletions) [9, 10]. In addition, for comparison, we fitted a regular decision tree model and Treesist-TB (labelled as “Single optimized Tree”) on aggregate datasets. The depth of the regular decision tree was set by 5-fold cross-validation up to a maximum of 15.

### Packages

The pipeline was implemented in Python (v3.7), building on the tree algorithm from sklearn (v0.23.1). The plausibility of putatively causal genomic variants identified was assessed using *Mycobrowser* [24].

## Supplementary Information

**Additional file 1.**

## Data Availability

The raw whole genome sequencing data is available from the European Nucleotide Archive (ENA) (see [18]). Computing code is available at https://github.com/WDee/Treesist-TB.
