# Supplementary material for: A modified decision tree approach to improve the prediction and mutation discovery for drug resistance in Mycobacterium tuberculosis
Source: BMC Genomics. 2022 Jan 11;23:46. doi: 10.1186/s12864-022-08291-4 (PMC8753810; doi:10.1186/s12864-022-08291-4)
Supplement: Supplementary file 1 — Additional file 1. [file 12864_2022_8291_MOESM1_ESM.docx]

**S1 Figure**

**Heatmap of included variant features in different sub-studies with colour-coding for subset of shared variants.** The studies are highlighted on the x-axis. The y-axis has format of (gene, genomic position). The darker green colour are genomic variants that are in genes that were detected more than once across different studies (i.e. the gene has two or more filled cells in different columns in the diagram)

**
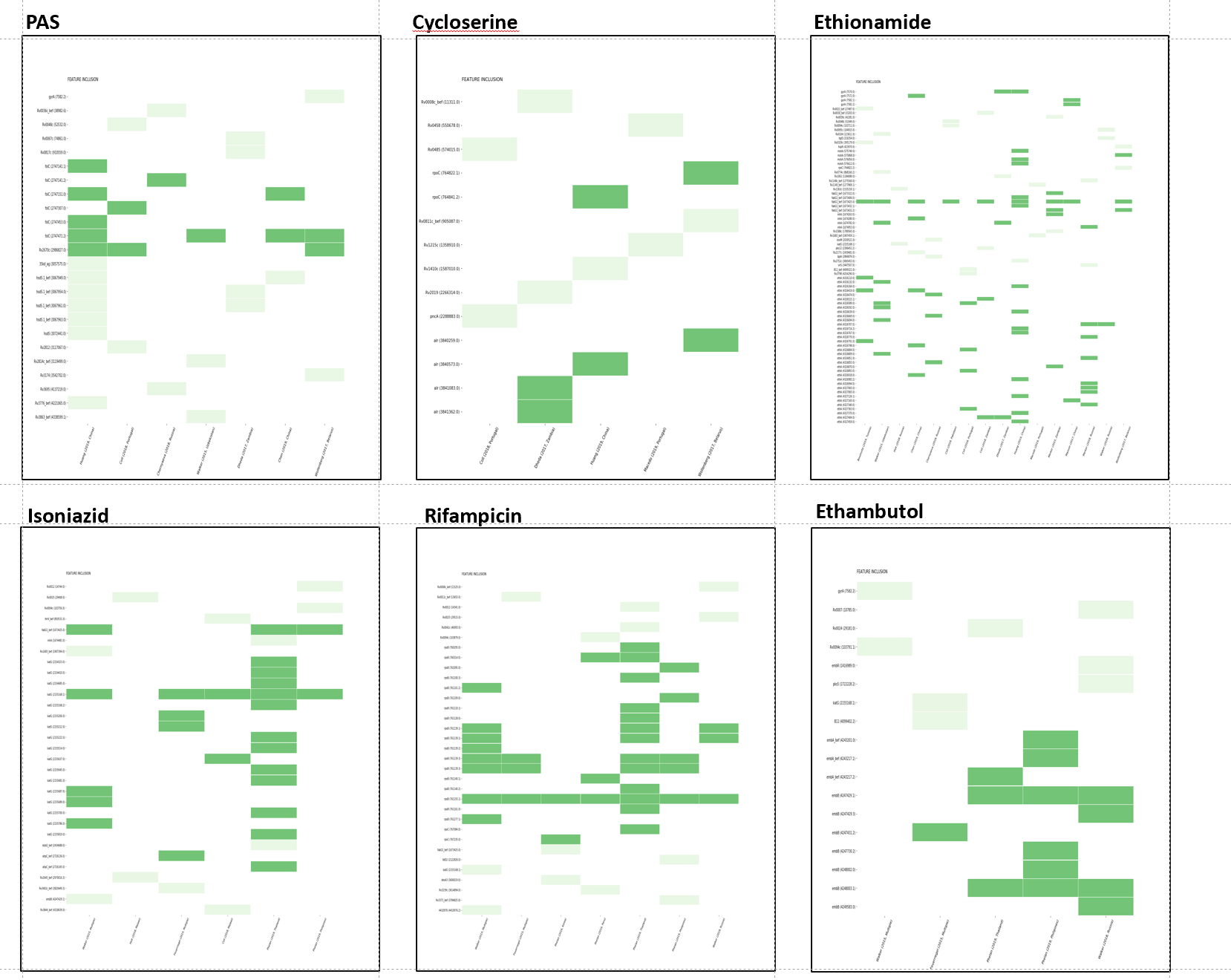
**

**S2 Figure**

**Tree diagrams of regular classification tree (left) and Treesist-TB (right).** The nodes in the trees are colour coded as blue (resistant) and orange (susceptible). Each node indicates the splitting variable, the improvement in purity (Gini), the total number of samples and the split over the left and the right nodes

**A) Isoniazid**

**
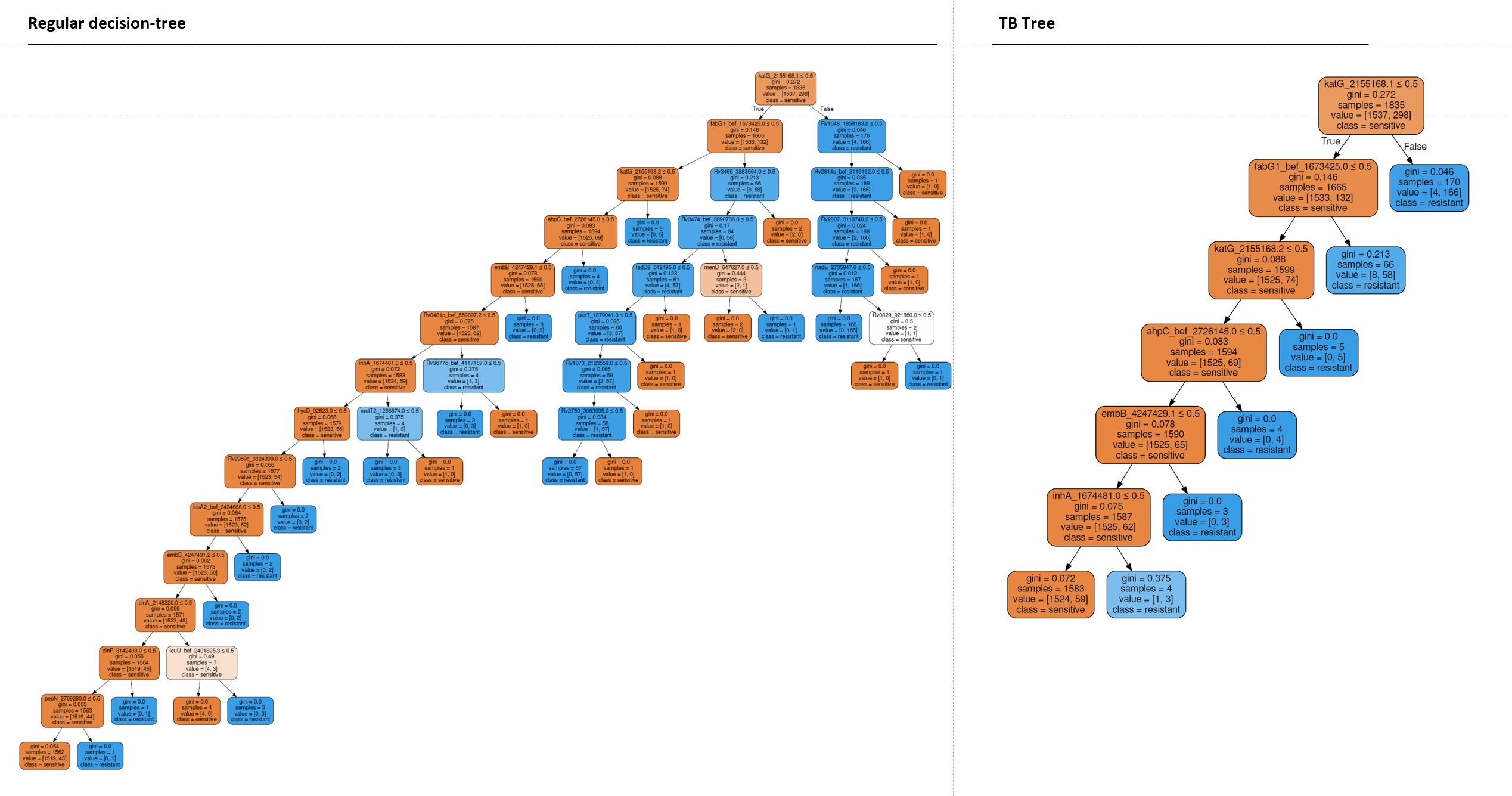
**

**B) Rifampicin**

**
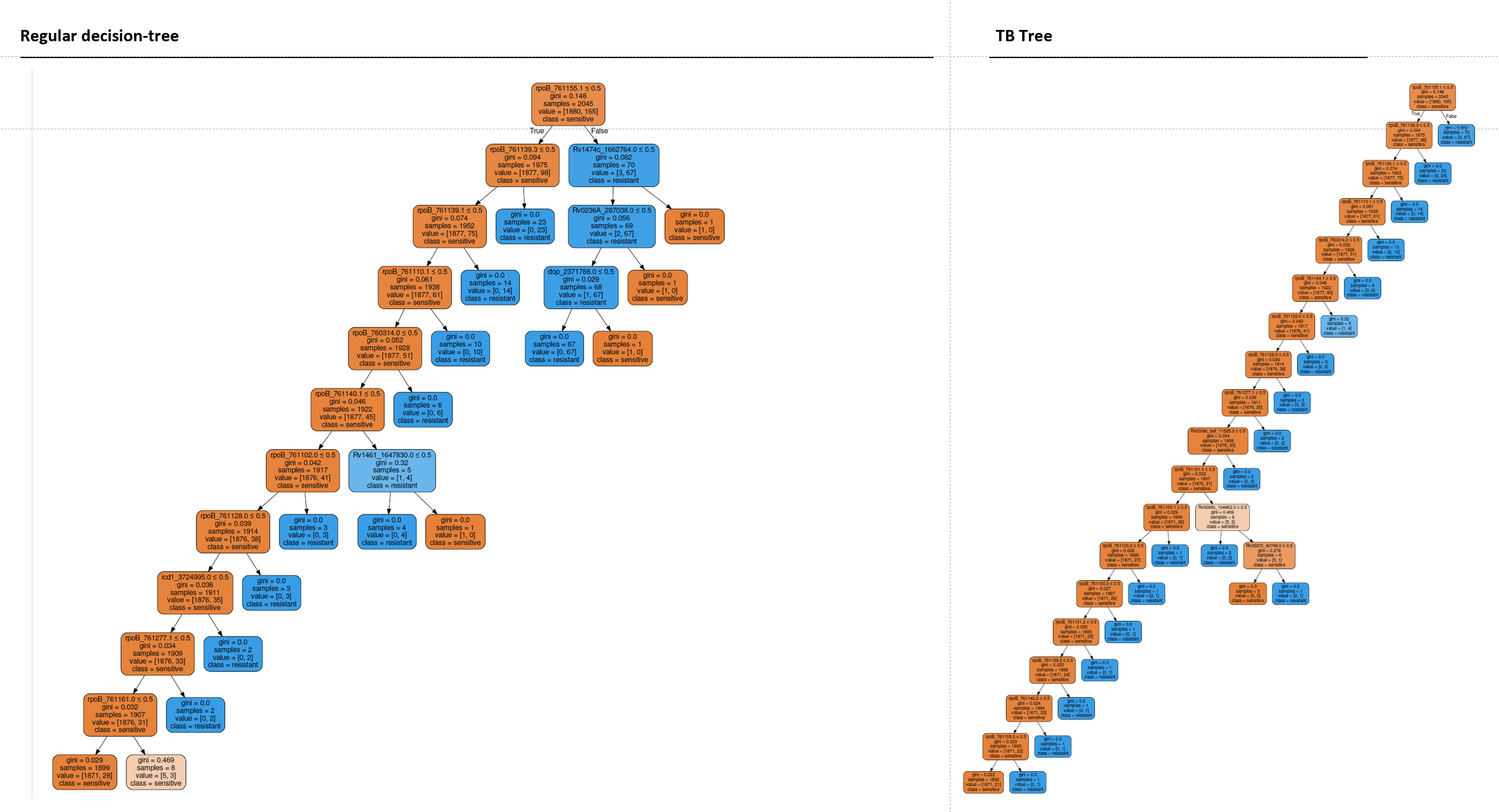
**

**C) Ethambutol**

**
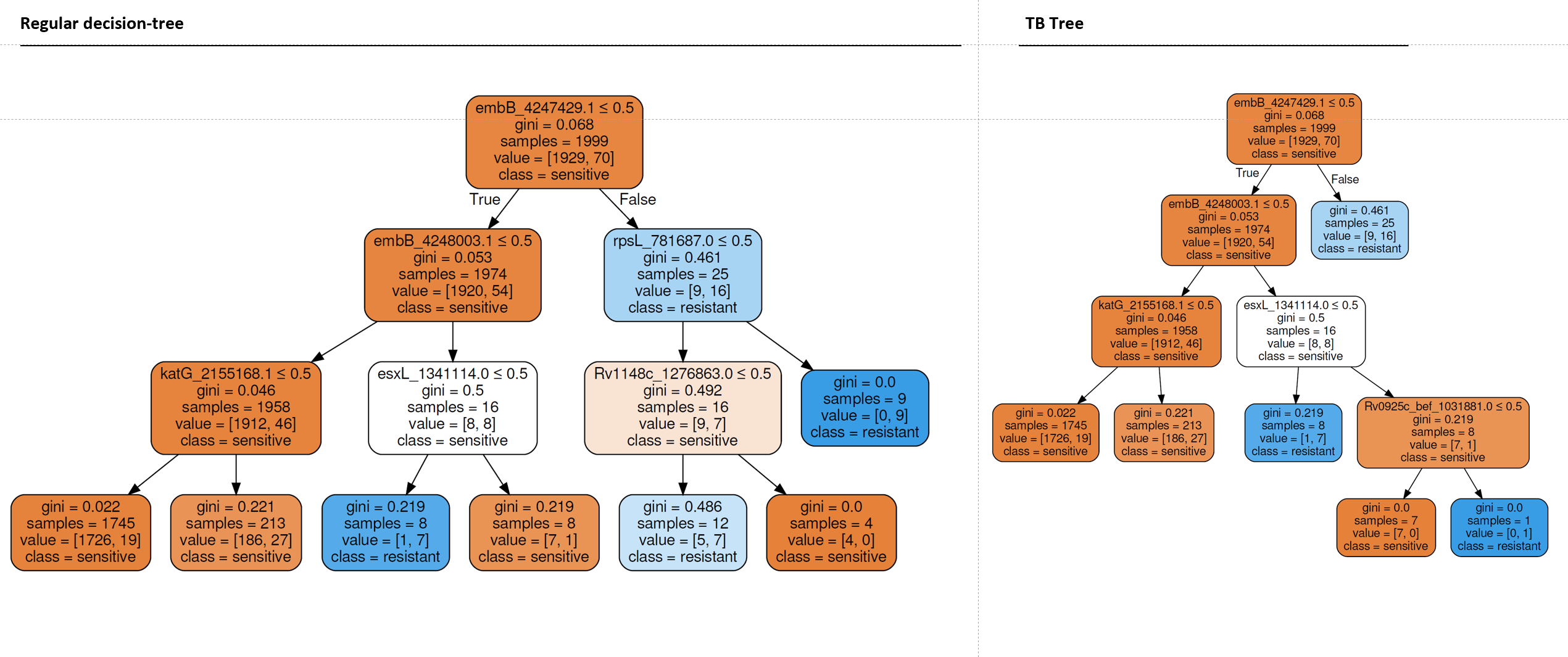
**

**S3 Figure**

**Tree diagrams of regular classification tree (left) and Treesist-TB (right).** The nodes in the trees are colour coded as blue (resistant) and orange (susceptible). Each node indicates the splitting variable, the improvement in purity (Gini), the total number of samples and the split over the left and the right nodes

1. **Para-aminosalisylic acid**

**
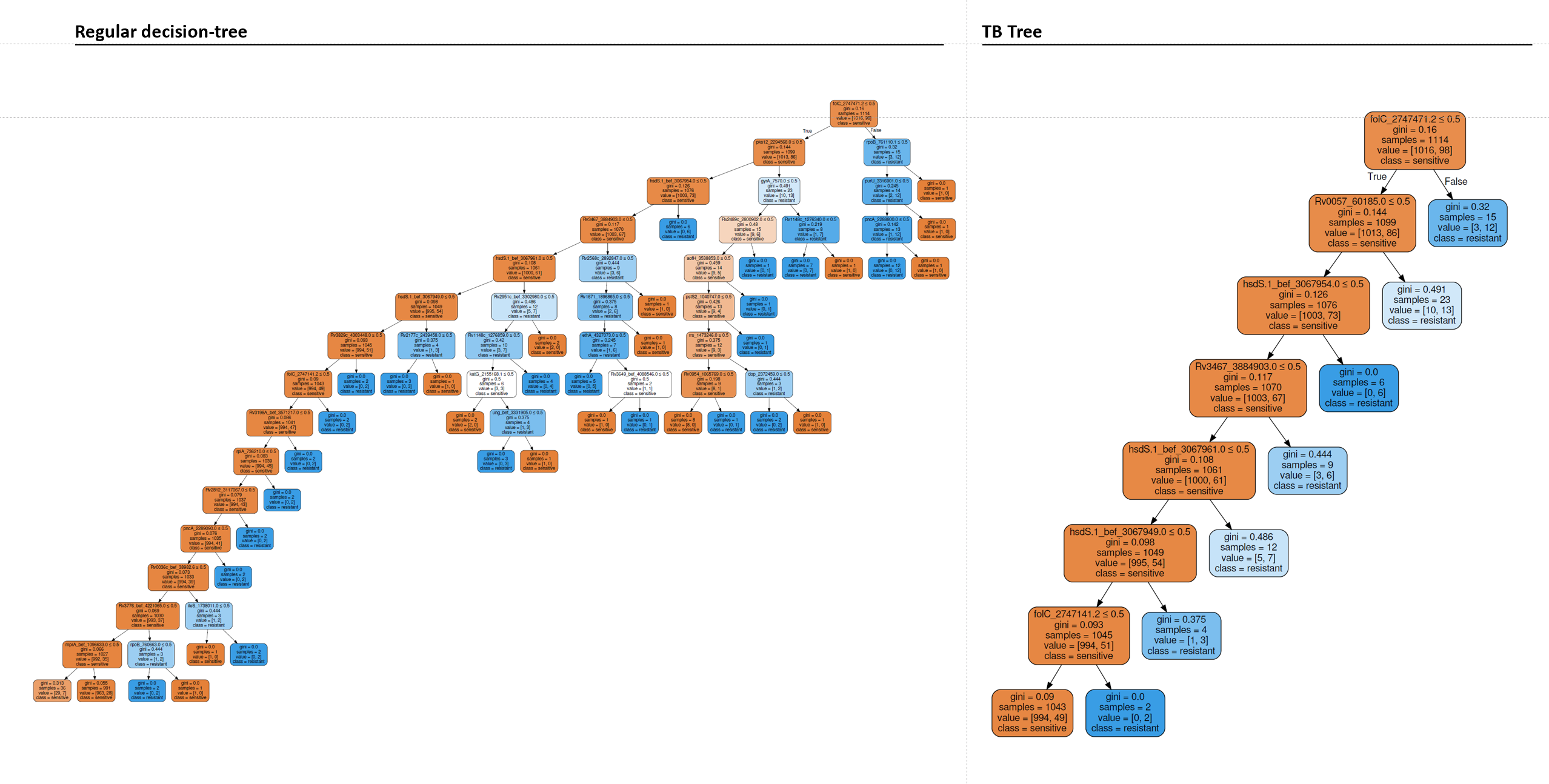
**

1. **Cycloserine**


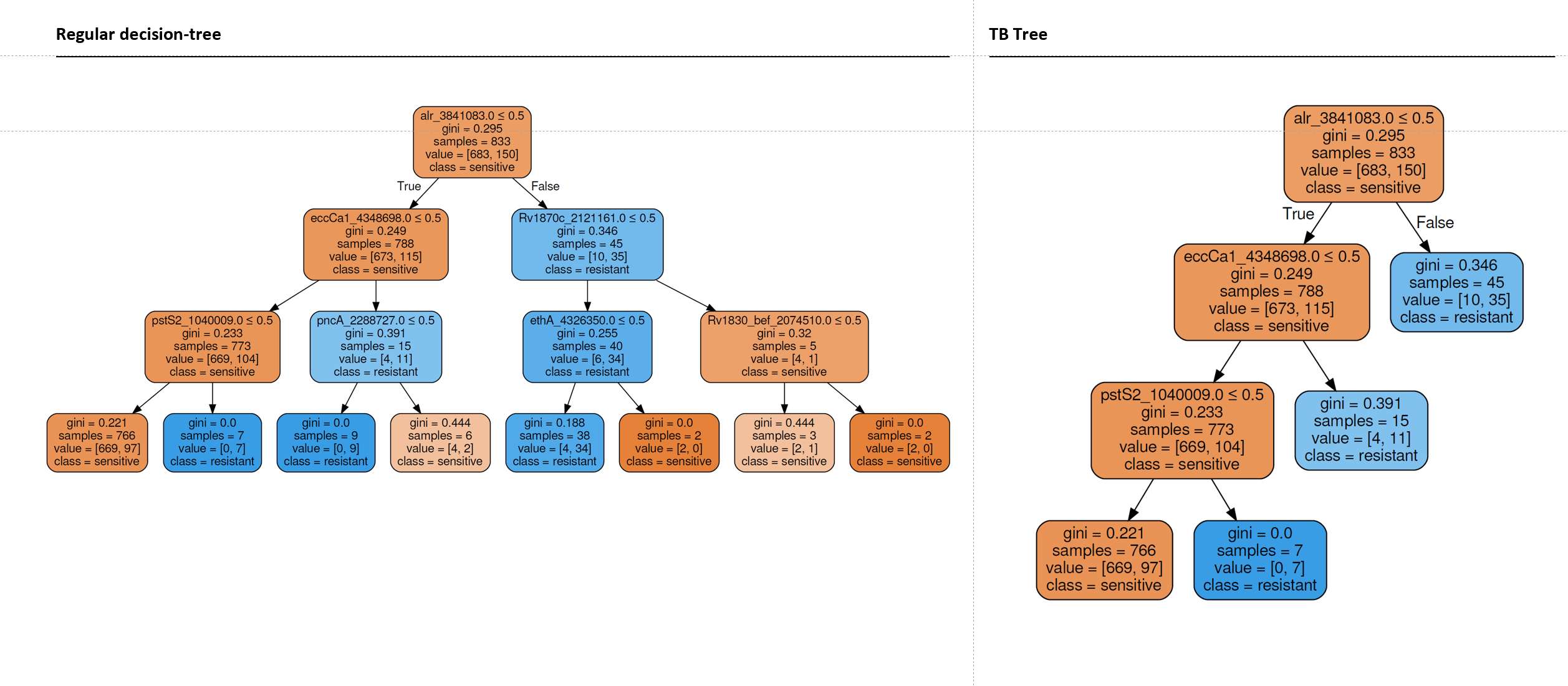


1. **Ethionamide**

**
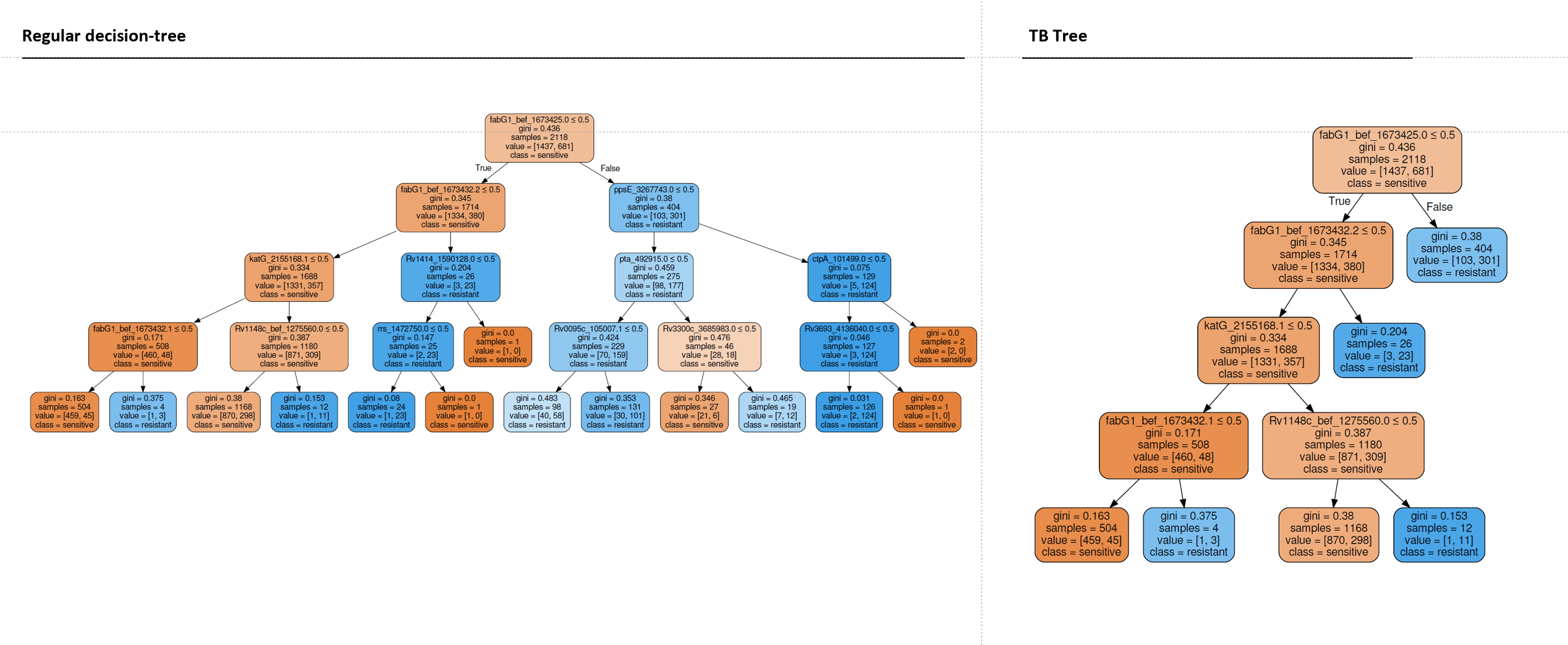
**

**S1 Table**

**Phenotypic drug susceptibility tests status by lineage**

| Lineage | N | % | Susc. | RR-TB | MDR-TB | XDR-TB | Other DR |
| --- | --- | --- | --- | --- | --- | --- | --- |
| 1 | 3155 | 9.6 | 2758 | 14 | 164 | 8 | 211 |
| 2 | 8260 | 25.2 | 5243 | 200 | 1913 | 473 | 431 |
| 3 | 3745 | 11.4 | 3053 | 19 | 439 | 37 | 197 |
| 4 | 16700 | 51.0 | 13686 | 191 | 1732 | 239 | 852 |
| 5 | 253 | 0.7 | 245 | 0 | 2 | 0 | 6 |
| 7 | 148 | 0.4 | 142 | 0 | 2 | 0 | 4 |
| 8 | 52 | 0.1 | 52 | 0 | 0 | 0 | 0 |
| 9 | 3 | 0 | 3 | 0 | 0 | 0 | 0 |
| Other | 373 | 1.1 | 283 | 0 | 3 | 0 | 87 |
| Total | 32689 | 100 | 25465 | 424 | 4255 | 757 | 1788 |
| (%) |  |  | (77.9%) | (1.3%) | (13.0%) | (2.3%) | (5.5%) |

RR-TB rifampicin resistant; MDR-TB is defined as resistance to isoniazid and rifampicin; XDR-TB is defined as MDR-TB, with additional resistance to a fluoroquinolone and second-line injectable drug (pre-2021 definition).

**S2 Table**

**Sources of sequence data, and drug resistance phenotypes**

| Drug | Total tests | # susc. | # resist. | % resist. | # studies | Study  countries | Lineage | PMID |
| --- | --- | --- | --- | --- | --- | --- | --- | --- |
| INH | 1835 | 1537 | 298 | 16.2 | 6 | Malawi, Philippines, Thailand | 1-6 | 26116186,25336729, 25854485,25336729,31234910,31243306 |
| RIF | 2045 | 1880 | 165 | 8.1 | 7 | Russia, Peru, South Korea, Philippines, Thailand | 1-6 | PMC3939361,26116186, 25854485, 31234910,31243306,27005572,27005572 |
| EMB | 1999 | 1929 | 70 | 3.5 | 5 | Russia, Philippines, Thailand | 1-6 | PMC3939361,26116186, 25854485, 31234910,31243306 |
| PAS | 1114 | 1016 | 98 | 8.8 | 7 | Portugal, South Africa, Uzbekistan, Russia, China, Belarus | 1-4 | 30321294,29358649,29460750,26116186,28109869,PMC6685394,27903602 |
| CYS | 833 | 683 | 150 | 18.0 | 5 | Portugal, China, Belarus, South Africa | 1-4 | 30321294,29358649,28109869,27903602,30948181 |
| ETN | 2118 | 1437 | 681 | 32.2 | 16 | Russia, Tunisia, China, Pakistan, Portugal, South Africa, Belarus | 1-4 | 30321294,29358649,29460750,26116186,28109869,PMC6685394,27903602,30948181,30789128,PMC3939361,29358649,29358649,26418737,23995137,PMC3939361,PMC3939361 |

Susc. Susceptible; INH = Isoniazid, RIF = Rifampicin, PAS=para-aminosalisylic acid, CYS=cycloserine, ETH=ethionamide, EMB = Ethambutol

**S3 Table**

**Summary of drugs and loci in TB-Profiler library**

| Drug | Locus | Gene | No.  variants* | TB-Profiler SNPs | TB-Profiler Indels |
| --- | --- | --- | --- | --- | --- |
| Rifampicin | Rv0667 | *rpoB* | 115 | 94 | 25 |
|  | Rv0668 | *rpoC* | 92 | 8 | - |
| Isoniazid | *Rv1483* | *fabG1* | 22 | 11 | - |
|  | *Rv1484* | *inhA* | 22 | 13 | - |
|  | *Rv1908c* | *katG* | 93 | 226 | 37 |
|  | *Rv2245* | *kasA* | 16 | 4 | - |
|  | *Rv2428* | *ahpC* | 32 | 21 | - |
| Ethambutol | *Rv1267c* | *embR* | 34 | 20 | - |
|  | *Rv3793* | *embC* | 85 | 25 | - |
|  | *Rv3794* | *embA* | 112 | 9 | 6 |
|  | *Rv3795* | *embB* | 125 | 127 | 1 |
| Ethionamide | *Rv1483* | *fabG1* | 20 | 3 | - |
|  | *Rv1484* | *inhA* | 15 | 3 | - |
|  | *Rv3854c* | *ethA* | 208 | 33 | 42 |
|  | *Rv3855* | *ethR* | 25 | 2 | - |
| PAS | *Rv2447c* | *folC* | 23 | 18 | - |
|  | *Rv2671* | *ribD* | 7 | 1 | - |
|  | *Rv2754c* | *thyX* | 7 | 1 | - |
|  | *Rv2764c* | *thyA* | 25 | 19 | 5 |
| Cycloserine | *Rv2780* | *ald* | 54 | - | 12 |
|  | *Rv3423c* | *alr* | 22 | 3 | - |

* Number of genomic variants in the individual studies used

**S4 Table**

**Frequency of Treesist-TB inferred variants in rifampicin, isoniazid, and ethambutol across 32k *Mycobacterium tuberculosis* isolates**

| Drug | Gene | Mutation | TB-Profiler** | Susc. % | MDR-TB % | XDR-TB % | Other resist. % |
| --- | --- | --- | --- | --- | --- | --- | --- |
| RIF | *rpoB* | N163K | no | - | <0.1 | - | - |
| RIF | *rpoB* | V170F | Yes | - | 1.0 | <0.1 | 0.3 |
| RIF | *rpoB* | L430P | Yes | - | 1.7 | 1.3 | 0.7 |
| RIF | *rpoB* | Q432K | yes | - | 0.3 | - | 0.1 |
| RIF | *rpoB* | Q432L | yes | <0.1 | 0.3 | 0.3 | <0.1 |
| RIF | *rpoB* | D435Y | yes | - | 3.4 | 2.0 | 1.9 |
| RIF | *rpoB* | D435V | yes | - | 7.9 | 11.0 | 0.7 |
| RIF | *rpoB* | S441L | yes | - | 0.6 | 0.3 | 0.1 |
| RIF | *rpoB* | H445D | yes | - | 4.1 | 1.8 | 0.9 |
| RIF | *rpoB* | H445N | yes | - | 1.3 | 0.4 | 0.4 |
| RIF | *rpoB* | H445Y | yes | - | 5.5 | 2.5 | 2.2 |
| RIF | *rpoB* | H445R | yes | - | 2.1 | 0.9 | 0.2 |
| RIF | *rpoB* | H445L | yes | - | 1.4 | 0.8 | 0.2 |
| RIF | *rpoB* | S450L | yes | <0.1 | 65.3 | 70.7 | 4.9 |
| RIF | *rpoB* | L452P | yes | - | 2.9 | 5.9 | 0.6 |
| RIF | *rpoB* | I491F | yes | - | 1.4 | 0.6 | 0.6 |
| RIF | *rpoC* | N1239D | no | <0.1 | - | - | - |
| RIF | *rpoC* | E1289A | no | <0.1 | - | - | - |
| INH | *fabG1* | -126G>A | no | <0.1 | 16.8 | 34.6 | 12.6 |
| INH | *katG* | Y597D | no | - | - | - | <0.1 |
| INH | *katG* | T568P | no | <0.1 | <0.1 | - | - |
| INH | *katG* | A476V | no | <0.1 | - | - | - |
| INH | *katG* | S315T | yes | <0.1 | 79.2 | 78.6 | 28.8 |
| INH | *katG* | S315N | yes | - | 1.8 | 1.3 | 1.1 |
| INH | *katG* | S302R | yes | - | <0.1 | <0.1 | 0.1 |
| INH | *katG* | W300C | no | - | - | - | <0.1 |
| INH | *katG* | G297V | yes | <0.1 | <0.1 | - | <0.1 |
| INH | *katG* | P193fs | no | - | - | - | <0.1 |
| INH | *katG* | L159F | no | <0.1 | - | - | - |
| INH | *katG* | G156D | no | - | <0.1 | - | - |
| INH | *katG* | A144V | no | <0.1 | - | - | - |
| INH | *katG* | D142G | no | <0.1 | <0.1 | - | <0.1 |
| INH | *katG* | L141F | yes | <0.1 | <0.1 | - | 0.1 |
| INH | *katG* | N138D | yes | - | <0.1 | - | <0.1 |
| INH | *katG* | A109V | yes | - | <0.1 | - | <0.1 |
| INH | *katG* | Y98C | no | <0.1 | <0.1 | - | 0.2 |
| INH | *ahpC* | -4359G>A | no | - | 0.4 | - | <0.1 |
| INH | *ahpC* | -48G>A | yes | - | 1.2 | 1.2 | 0.4 |
| EMB | *embA* | -31delC | no | - | 0.2 | <0.1 | - |
| EMB | *embA* | -16C>T | yes | - | 2.1 | 4.4 | 0.2 |
| EMB | *embA* | -16C>A | no | <0.1 | 0.8 | 0.7 | <0.1 |
| EMB | *embB* | M306V | yes | - | 23.8 | 35.3 | 1.6 |
| EMB | *embB* | M306L | yes | - | 1.3 | 1.2 | 0.3 |
| EMB | *embB* | M306I | yes | <0.1 | 20.2 | 26.7 | 3.0 |
| EMB | *embB* | G406A | yes | - | 6.6 | 6.5 | 0.4 |
| EMB | *embB* | Q497K | yes | - | 1.2 | 0.9 | 0.3 |
| EMB | *embB* | Q497R | yes | - | 5.6 | 8.0 | 0.5 |
| EMB | *embB* | D1024N | yes | - | 2.0 | 1.8 | 0.1 |

* from [24]; INH = Isoniazid, RIF = Rifampicin, EMB = Ethambutol; RR-TB rifampicin resistant; MDR-TB multidrug resistant; XDR-TB Extensively drug resistant; ** underlined if mentioned in [www.who.int/publications/i/item/9789240028173](http://www.who.int/publications/i/item/9789240028173) as a high confidence (group 1) resistance mutation (sourced Nov. 2021)

**S5 Table**

**Frequency of Treesist-TB inferred variants in para-aminosalisylic acid, cycloserine, and ethionamide across 32k *Mycobacterium tuberculosis* isolates***

| Drug | Gene | mutation | TB-Profiler** | Susc. % | MDR-TB % | XDR-TB % | Other resist. % |
| --- | --- | --- | --- | --- | --- | --- | --- |
| PAS | *folC* | E153G | yes | - | 0.3 | 0.4 | 0.0 |
| PAS | *folC* | E153A | yes | - | 0.2 | 0.3 | <0.1 |
| PAS | *folC* | S150G | yes | - | 0.9 | 1.4 | 0.3 |
| PAS | *folC* | S98G | no | - | 0.0 | 0.3 | 0.0 |
| PAS | *folC* | R49Q | no | - | 0.8 | 0.3 | 0.2 |
| PAS | *folC* | I43T | yes | - | 0.7 | 3.1 | 0.2 |
| PAS | *Rv2670c* | A5V | no | <0.1 | 4.5 | 6.1 | 0.8 |
| PAS | *thyX* | -4C>T | no | <0.1 | 0.4 | 1.7 | <0.1 |
| PAS | *thyX* | -9G>A | no | <0.1 | 0.6 | 0.5 | 0.1 |
| PAS | *thyX* | -16C>T | yes | <0.1 | 1.8 | 3.6 | 0.5 |
| PAS | *thyX* | -18G>T | no | - | <0.1 | 0.2 | <0.1 |
| CYS | *rpoC* | D485Y | no | - | 0.5 | 1.5 | <0.1 |
| CYS | *rpoC* | I491T | yes | - | 1.3 | 4.3 | <0.1 |
| CYS | *alr* | Y388D | no | - | 0.5 | 1.1 | - |
| CYS | *alr* | L283P | no | <0.1 | - | - | - |
| CYS | *alr* | L113R | yes | - | 0.8 | 8.5 | <0.1 |
| CYS | *alr* | T20M | no | - | 0.1 | 0.4 | <0.1 |
| ETH | *gyrA* | A90V | yes | <0.1 | 4.6 | 32.0 | 1.4 |
| ETH | *gyrA* | S91P | yes | - | 0.9 | 8.7 | 0.6 |
| ETH | *gyrA* | D94A | yes | <0.1 | 2.3 | 12.8 | 0.4 |
| ETH | *gyrA* | D94G | yes | <0.1 | 5.9 | 36.5 | 2.3 |
| ETH | *mshA* | A133fs | no | - | <0.1 | - | - |
| ETH | *mshA* | H175fs | no | - | <0.1 | - | - |
| ETH | *mshA* | V237L | no | - | <0.1 | - | - |
| ETH | *mshA* | A422V | no | - | <0.1 | - | - |
| ETH | *fabG1* | -23G>C | no | - | <0.1 | - | - |
| ETH | *fabG1* | -107G>A | no | <0.1 | 0.4 | 1.3 | <0.1 |
| ETH | *fabG1* | -126G>A | no | <0.1 | 16.8 | 34.6 | 12.6 |
| ETH | *fabG1* | -133A>G | no | - | 1.3 | 4.2 | 0.8 |
| ETH | *fabG1* | -133A>T | no | - | 1.4 | 5.7 | 0.3 |
| ETH | *inhA* | I21T | yes | - | 1.2 | 1.0 | 0.3 |
| ETH | *inhA* | R27W | no | - | <0.1 | - | - |
| ETH | *inhA* | I194T | yes | - | 2.0 | 5.3 | 0.3 |
| ETH | *inhA* | P251R | no | 1.3 | 1.6 | 1.4 | 1.1 |
| ETH | *ethA* | W455 | no | - | 0.1 | 0.6 | <0.1 |
| ETH | *ethA* | K448fs | no | - | 0.1 | <0.1 | <0.1 |
| ETH | *ethA* | P436fs | no | - | <0.1 | - | - |
| ETH | *ethA* | A352fs | no | - | 0.2 | 0.6 | 0.1 |
| ETH | *ethA* | P334A | no | 0.4 | 0.9 | 1.0 | 0.4 |
| ETH | *ethA* | F320S | no | - | - | <0.1 | - |
| ETH | *ethA* | L295fs | no | - | 0.2 | <0.1 | - |
| ETH | *ethA* | C294 | no | - | <0.1 | - | - |
| ETH | *ethA* | R279 | no | - | <0.1 | 0.2 | - |
| ETH | *ethA* | Q269 | yes | - | 0.2 | <0.1 | - |
| ETH | *ethA* | M260I | no | <0.1 | <0.1 | - | 0.1 |
| ETH | *ethA* | W256 | no | <0.1 | 0.6 | 2.0 | 0.1 |
| ETH | *ethA* | C253F | no | - | - | 0.2 | - |
| ETH | *ethA* | T236fs | no | - | <0.1 | - | - |
| ETH | *ethA* | Y235fs | no | - | 0.2 | 0.6 | <0.1 |
| ETH | *ethA* | W228 | no | <0.1 | - | <0.1 | - |
| ETH | *ethA* | N226fs | no | <0.1 | <0.1 | <0.1 | - |
| ETH | *ethA* | K224 | no | <0.1 | <0.1 | - | - |
| ETH | *ethA* | A222V | no | - | <0.1 | - | - |
| ETH | *ethA* | S208L | no | <0.1 | <0.1 | - | - |
| ETH | *ethA* | R207G | yes | - | <0.1 | 0.2 | <0.1 |
| ETH | *ethA* | V202F | no | - | <0.1 | 0.2 | - |
| ETH | *ethA* | L194P | no | - | <0.1 | - | - |
| ETH | *ethA* | T186P | no | - | <0.1 | <0.1 | <0.1 |
| ETH | *ethA* | P164R | no | - | <0.1 | - | - |
| ETH | *ethA* | P160fs | no | - | 0.4 | <0.1 | 0.2 |
| ETH | *ethA* | C137R | no | - | 0.3 | 0.2 | <0.1 |
| ETH | *ethA* | C137R | no | - | 0.3 | 0.2 | <0.1 |
| ETH | *ethA* | W116 | no | <0.1 | <0.1 | 0.3 | - |
| ETH | *ethA* | K103fs | no | - | <0.1 | - | - |
| ETH | *ethA* | W45 | no | - | 0.1 | 0.6 | <0.1 |
| ETH | *ethA* | K37fs | no | - | 1.4 | 1.8 | 0.1 |
| ETH | *ethA* | L35R | no | - | 0.2 | - | - |
| ETH | *ethA* | Q24 | no | <0.1 | 0.8 | 0.9 | 0.1 |
| ETH | *ethA* | D6fs | no | - | <0.1 | - | - |

* from [24]; - refers to a frequency of zero; PAS=para-aminosalisylic acid, CYS=cycloserine, ETH=ethionamide; RR-TB rifampicin resistant; MDR-TB multidrug resistant; XDR-TB Extensively drug resistant; ** underlined if mentioned in [www.who.int/publications/i/item/9789240028173](http://www.who.int/publications/i/item/9789240028173) as a high confidence (group 1) resistance mutation (sourced Nov. 2021)
